# The Effect of 90 and 120 Min of Running on the Determinants of Endurance Performance in Well‐Trained Male Marathon Runners

**DOI:** 10.1111/sms.70076

**Published:** 2025-05-15

**Authors:** Michele Zanini, Jonathan P. Folland, Richard C. Blagrove

**Affiliations:** ^1^ School of Sport, Exercise, and Health Sciences Loughborough University Loughborough UK; ^2^ National Institute for Health and Care Research (NIHR), Leicester Biomedical Research Centre Leicester UK

**Keywords:** durability, endurance performance, fatigue, marathon, physiological determinants, physiological resilience, prolonged running

## Abstract

The combination of maximal oxygen uptake (V̇O_2_max), fractional utilization at lactate threshold (FU_LT_), and running economy (RE) is considered to largely determine/predict marathon performance, which is also closely associated with the speed at lactate threshold (sLT). Although these determinants are considered to deteriorate during prolonged running, except for RE, their temporal changes with fatigue remain largely unknown. This study aimed to measure the changes in V̇O_2_max, FU_LT_, RE, and sLT after running for 90 and 120 min in the heavy‐intensity domain. Fourteen trained marathon runners (V̇O_2_max 63.1 ± 5.8 mL·kg^−1^·min^−1^; marathon time 2:46:58 h:mm:ss) completed three separate visits to determine sLT, FU_LT_, and V̇O_2_peak in the following conditions (sessions): unfatigued, and after two prolonged runs of 90 and 120 min at a fixed speed (10% Δ between LT and lactate threshold 2). During the runs, respiratory gases were collected at 15 min intervals to quantify RE. Decreases in V̇O_2_peak (−3.1%, *p* = 0.04 [post‐90]; −7.1%, *p* < 0.001 [post‐120]) and subsequent increases in FU_LT_ (+2.8%, *p* = 0.03 [post‐90]; +4.9% *p* = 0.01 [post‐120]) both occurred at an increasing rate with run duration, with FU_LT_ changes linked to the decreased V̇O_2_peak, while RE (mL·kg^−1^·km^−1^) deteriorated more linearly with time (by 4.2% [post‐90] and 5.8% [post‐120], *p* < 0.001). sLT also showed a nonlinear decrease, from 14.0 to 13.5 (*p* = 0.01 post‐90), to 13.0 km·h^−1^ (*p* < 0.001 post‐120). In conclusion, performance determinants and sLT changed following 90 min, and particularly 120 min of prolonged running. These dynamic changes have strong implications for running performance and would particularly affect longer duration events such as the marathon.

## 1 Introduction

The marathon is a popular endurance running event, with more than one million people completing the distance every year. The classical model of endurance running suggests that marathon speed can be predicted, physiologically, from a runner's “performance V̇O_2_”, which is given by maximal oxygen uptake (V̇O_2_max) and fractional utilization of V̇O_2_max at lactate threshold (FU_LT_), and the subsequent conversion of metabolic energy into running speed, described by their running economy (RE) [1]. In Joyner's model, these three physiological factors are used to calculate the speed at lactate threshold (sLT; defined as the speed eliciting an increase in blood lactate (BLa) above baseline values), which is closely associated with marathon performance speed [2, 3]. Although Joyner's model can be used to estimate endurance running performance, one limitation is that it does not account for the temporal changes in these three determinants that result from fatigue during prolonged exercise. The capacity to minimize the detrimental physiological changes during prolonged exercise has recently been suggested to be a fourth independent determinant of endurance performance, termed “resilience” or “durability” [4, 5]. Specifically, durability describes the time of onset and magnitude of the deterioration in performance and its determinants during or following prolonged exercise [5]. Indeed, there is a growing body of evidence indicating that endurance performance determinants are not static and change during prolonged exercise [6, 7, 8]. Although the changes in RE have been investigated during and after prolonged running [8, 9, 10, 11], the changes that may occur in V̇O_2_max and FU_LT_ due to running‐induced fatigue are largely unknown, and the impact of these changes on sLT, closely related to marathon speed, has not been investigated.

Only two studies to date have documented changes in V̇O_2_max following prolonged running in trained athletes, with no change reported after 60 min at 70% V̇O_2_max [12], but a 6% decrease after a half‐marathon race (~90 min) [13]. Therefore, the V̇O_2_max changes during prolonged running could be dependent on exercise duration, although this has not yet been investigated. If V̇O_2_max is reduced by prolonged exercise, this may negatively impact marathon performance due to the increase in relative intensity needed to maintain a given speed. Furthermore, a reduced V̇O_2_max may limit the ability to produce high‐intensity efforts in the final stages of a race. It is also of interest to investigate if changes in V̇O_2_max follow a similar pattern of deterioration to those observed for sub‐maximal exercise intensities (i.e., RE) [11], or whether a different duration dependency exists.

FU_LT_, often used synonymously with sLT, is an index of the boundary between moderate and heavy exercise intensity domains [14, 15]. To our knowledge, changes after prolonged running have not been documented, and only one study has attempted to describe the changes in FU_LT_ following prolonged cycling, reporting a 5% decrease after 150 min of exercise [7]. However, the end exercise FU_LT_ in that study was estimated based on pre‐trial V̇O_2_max values, as the measurement of V̇O_2_max was not repeated after exercise, and thus any changes in V̇O_2_max could confound the estimation of end exercise FU_LT_. Therefore, measuring the changes in FU_LT_ alongside V̇O_2_max after prolonged exercise is required to accurately document the changes in these two key performance determinants. Despite no research directly investigating the dynamic alterations in FU_LT_ in response to exercise, duration‐dependent changes in the power output corresponding to metabolic thresholds have been investigated in cycling [6, 7, 16, 17]. For intensities corresponding to LT, an 8%–10% decrease in power has been observed following trials of 2–3 h [7, 16]. Similarly, decreases in critical power have been found after 2 h of cycling [6, 17, 18] and after expanding 2000–3000 Kcal in elite riders [19, 20]. Changes in metabolic thresholds following prolonged cycling have also been found to depend on exercise duration, with longer trials leading to a larger downward drift in critical power or ventilatory threshold (VT1) [17, 21]. It is therefore of interest to understand the time course of the deterioration in FU_LT_ after prolonged running alongside the other determinants of performance, as well as assess changes in sLT, given its close association to marathon speed.

In contrast to V̇O_2_max and FU_LT_, the deterioration in RE with prolonged running has been well documented [8, 10, 22, 23], with measurable increases occurring after about 60 min in well‐trained runners [11] and changing by 7%–16% at the end of marathon races [8, 9, 10]. The wide range of RE decrements between studies can be partially explained by trial intensity (higher intensity leads to greater decrement) [22] and athletes' performance level (higher standard leads to smaller decrement) [11]. For well‐trained runners, marathon races typically occur in the heavy‐intensity domain [2, 24], thus evaluating the changes in the physiological determinants after exercise at this intensity would have the greatest validity. Although RE can be measured during prolonged exercise at the trial speed, assessing sLT, FU_LT_, and V̇O_2_max requires distinct incremental exercise tests in the fresh and fatigued conditions, which may explain the limited data available on the changes in these variables. Since incremental tests induce fatigue, conducting them before or during a prolonged run would confound the effects of the running trial being investigated. An alternative approach is to perform the incremental tests on different test days (i.e., when unfatigued, and after different durations of prolonged running), which can be combined to generate a temporal profile.

If V̇O_2_max, FU_LT_, and RE dynamically change during prolonged running, this has important consequences for race performance and modeling, particularly for the marathon [4]. For example, if RE deteriorates (increasing sub‐max V̇O_2_) and V̇O_2_max declines, then FU at marathon intensity would rise, making the exercise increasingly unsustainable. Therefore, quantifying the magnitude of changes in the physiological determinants of performance during prolonged running and their duration dependency may provide further insight into the limitations of endurance performance. Moreover, if deterioration rates are known, this may help develop effective training and racing strategies, e.g., for athletes to pace themselves and time their final effort in a race scenario.

The primary aim of this study was to quantify and compare the changes in running performance determinants (RE, V̇O_2_max, and FU_LT_) as well as sLT following prolonged running in the heavy‐intensity domain of well‐trained male marathon runners. Measurements were taken on three separate occasions (conditions): unfatigued, after 90 min, and after 120 min of running, to provide a temporal profile of the changes. It was hypothesized that performance determinants and sLT would change following the prolonged runs, but at differing rates, due to diversities in the physiology underpinning each of them.

## 2 Methods

### 2.1 Participants

Seventeen male endurance runners volunteered and gave written informed consent to participate in this study, which was approved by the Loughborough University Ethics Sub‐Committee. To be eligible to take part, participants had to be aged 18–40 years, have run a marathon in < 3 h in the previous 6 months, be currently running ≥ 40 km·week^−1^, completing runs of ≥ 105 min in duration ≥ 2 times·month^−1^, and free of musculoskeletal injury. Participants completed a 4‐week training questionnaire and recorded their recent (past 6 months) race performances.

#### 2.1.1 Experimental Overview

Participants visited the laboratory on three occasions, with successive visits 3–7 and 7–14 days apart. Participants were asked to refrain from any caffeine and alcohol ingestion, and intense exercise in the 24 h preceding each trial. All testing took place in the same physiology laboratory on a motorized treadmill (3DI, Treadmetrix, Utah, US), and participants completed the three visits at the same time of the day. The laboratory conditions were noted before each trial using a portable weather station (WS6730, Technoline, Germany) and were similar for all visits (temperature 18°C–21°C, relative humidity 45%–55%). Participants recorded their diet and exercise in the 48 h preceding Visit 1 and were then asked to replicate these prior to the second and third trials for standardization. The first visit consisted of a discontinuous incremental treadmill running assessment (step test) to evaluate speed at LT and lactate threshold 2 (LT2), and following a short rest by a continuous incremental test to quantify V̇O_2_max. In the second and third visits, participants were required to perform a 90 and 120 min run (in a randomized‐counter balanced order) at a speed corresponding to 10% delta between LT and LT2. Following both prolonged runs, participants repeated the step test and maximal ramp test using the same protocol as Visit 1. Participants were required to wear the same footwear for all trials, and advanced footwear technology was not permitted due to its enhancing effect on RE [25].

### 2.2 Visit 1: Lactate Thresholds and V̇O_2_max

Upon arrival at the laboratory, participants' height and body mass (BM) were measured on a stadiometer (Harpenden Stadiometer, Holtain Ltd., UK) and weighing scale (Seca 700; Seca Hamburg, Germany). Participants then completed a 5 min warm‐up at a self‐selected speed before performing a discontinuous incremental step test on a treadmill consisting of 7–10 stages of 3 min, at speed increments of 1 km·h^−1^ per stage, until volitional exhaustion was reached [26]. Each stage was interspersed with a 30 s recovery to allow collection of a capillary blood sample from the earlobe for determination of BLa. The speed of the first stage was determined using participants' best race times and their physiological response to the warm‐up period, and the testing ended when the rating of perceived exhaustion (RPE) was > 18. The treadmill incline was kept at 1% to account for differences in air resistance compared to overground running [27]. RPE using the Borg 6–20 scale was measured, and heart rate (HR) was continuously recorded via a chest strap (H10, Polar, Kempele, FIN). LT, defined as the first rise in BLa from baseline, was calculated using a log–log analysis [28], while LT2 was calculated via the modified log–log Dmax method [29]. After the incremental step test, participants passively rested for 5 min before commencing the continuous maximal ramp test. The speed was set at 2 km·h^−1^ slower than the final speed reached on the discontinuous test, with the test commencing at a gradient of 1%. Every minute thereafter, the incline increased by 1% until volitional exhaustion was reached despite strong verbal encouragement to continue. Maximal HR (HR max) was considered the highest HR recorded during the step or ramp test. Throughout both tests, participants wore a low‐dead‐space mask and breathed through an impeller turbine assembly (Jaeger Triple V, Jaeger GmbH, Hoechberg, Germany) to measure gas composition in inspired and expired air via an open circuit metabolic cart (Jaeger Vyntus CPX, Carefusion, San Diego, CA). The inspired and expired‐gas volume and concentration signals were continuously sampled, the latter using paramagnetic (O_2_) and infrared (CO_2_) analyzers (Jaeger Vyntus CPX, Carefusion, San Diego, CA) via a capillary line. These analyzers were calibrated before each test using a known gas mixture (16% O_2_ and 5% CO_2_) and ambient air. The turbine volume transducer was calibrated using a 3‐L syringe (Hans Rudolph, KS). The volume and concentration signals were time aligned, accounting for the transit delay in capillary gas and analyzer rise time relative to the volume signal.

#### 2.2.1 Visit 2 and 3: 90 and 120 Min Running Trial

**FIGURE 1:**
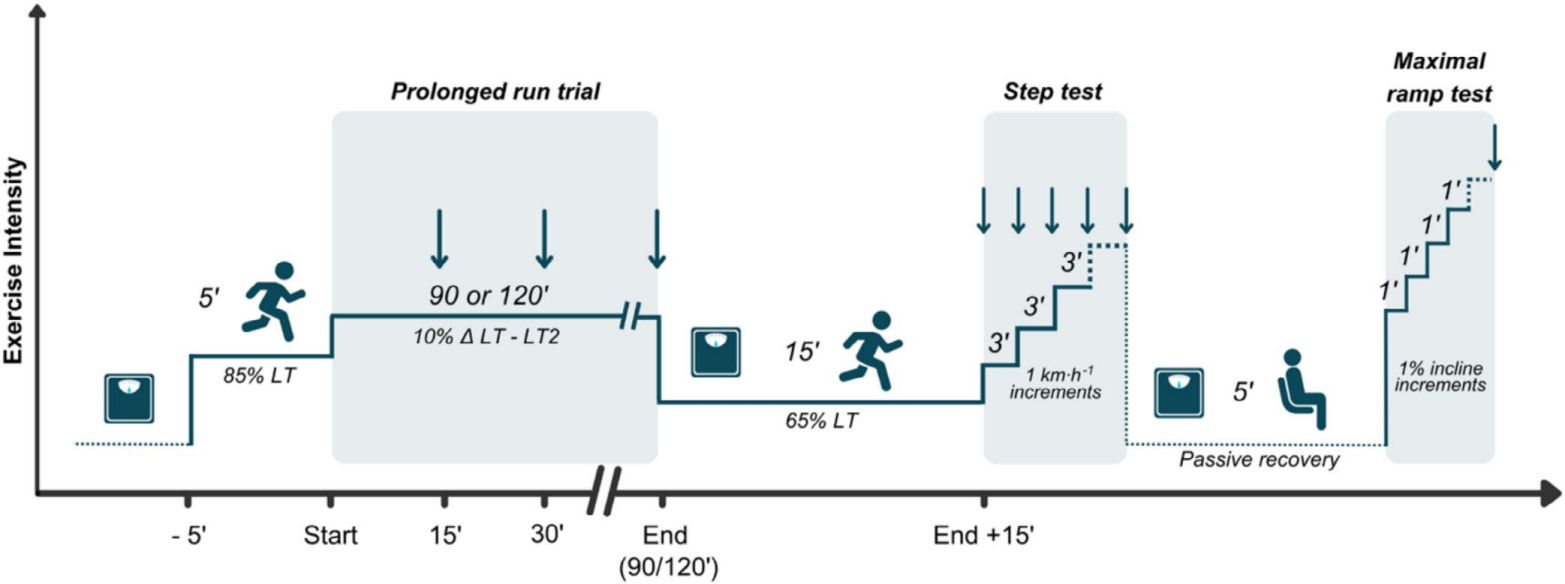
Graphical representation of the exercise testing protocol for the 90 and 120 min visits. The thin line indicates the exercise intensity during each stage of the protocol. The weighting scale icon indicates when BM was measured, and the darker background highlights the running test at each stage. Arrows indicate time points for physiological data collection (respiratory gases, BLa, HR, and RPE). See methods for the extensive details.

Upon arrival, the participant's BM was recorded. Participants were then asked to stand quietly on the treadmill for 5 min to record respiratory gases at rest, and subsequently performed a 5 min warm‐up at a speed corresponding to 85% of LT. Thereafter the speed was set to 10% delta between LT and LT2, and participants ran for either 90 or 120 min. Participants wore a mask throughout the test and respiratory gases were sampled discontinuously for 5 min every 15 min utilizing the same method and equipment described for Visit 1. Participants straddled the treadmill belt for a few seconds before and after each sampling period, to attach and detach the mouthpiece. HR, BLa, and RPE were also sampled at 15 min intervals, and a 30 mL carbohydrate (CHO) solution (25% concentration, to provide 30 g·h^−1^ of CHO) was given at the end of each sampling period, following which participants could drink water ad libitum. Two motorized fans were positioned approximately 2 m behind and in front of the participant, to provide a cooling effect throughout the trial and reduce the risk of hyperthermia. Following the prolonged run, participants' BM was measured, and they were asked to exercise at 65% of LT for 15 min to allow BLa to return to baseline level. Participants thereafter started the incremental step test, followed by another BM measure and by a maximal ramp test to exhaustion, reproducing the procedures described in Visit 1. The full prolonged run protocol is displayed in Figure 1.

### 2.3 Measurements

#### 2.3.1 Anthropometry, Heart Rate, and Blood Lactate

BM was measured using a digital scale to the nearest 0.1 kg, and height was recorded to the nearest 0.01 m using a stadiometer. HR was continuously recorded, and the average of the last 30 s of each stage was recorded. Capillary blood was collected via a 20‐μL capillary tube from the participant's ear lobe. The sample was immediately haemolysed and assessed for BLa concentration (Biosen C‐Line, EKF Diagnostics, Cardiff, UK).

#### 2.3.2 Maximal Measures

Breath‐by‐breath V̇O_2_ data were continuously recorded and initially filtered to exclude errant breaths, defined as values lying more than 4 standard deviations (SDs) from the local mean. Subsequently, the breath‐by‐breath data were converted to second‐by‐second data using linear interpolation. V̇O_2_max was defined as the highest 30 s moving average from the V̇O_2_ data. The presence of a V̇O_2_ plateau was also visually inspected and was considered to be attained if V̇O_2_ remained unchanged for > 30 s during the maximal ramp test. BM was recorded between the step test and the maximal ramp test and used to adjust V̇O_2_max relative to the appropriate BM. The speed corresponding to V̇O_2_max (sV̇O_2_max) was estimated by extrapolation of the linear regression line plotted between V̇O_2_ and speed recorded in the submaximal stages of the incremental step test, as long as RER was < 1.00 for the stage. These measurements were made in the three conditions: unfatigued, post 90, and post 120 min of running.

#### 2.3.3 Running Economy

During the prolonged runs, the average of V̇O_2_ and V̇CO_2_ data collected during the final 2 min of each 15 min stage were used to calculate the oxygen cost (OC) of running expressed as mL·kg^−1^·km^−1^, as a measure of RE. Energy cost (EC) was also calculated, and results are included in the online resources (Table S1), as it has been recently demonstrated that negligible differences exist between EC and OC when changes are measured throughout prolonged exercise [11]. Briefly, updated nonprotein respiratory quotient equations [30] were used to estimate substrate utilization (g·min^−1^). The energy derived from each substrate was calculated by multiplying fat and carbohydrate utilization by 9.75 and 4.07 kcal, respectively [31]. Absolute EC was calculated as the sum of the energy derived from fat and carbohydrate expressed as kcal·kg^−1^·km^−1^. To account for changes in BM during the prolonged runs, BM pre‐ to post‐trial was measured and linear regression was used to estimate BM at each measurement time point. RE was then calculated based on the adjusted BM for each time point.

#### 2.3.4 Fractional Utilization During the Prolonged Run

Fractional utilization of V̇O_2_max in mL·kg^−1^·min^−1^ (FU_run_) during the prolonged run was measured to assess the effect of prolonged exercise at a speed close to the participant's marathon performance. The intensity was calculated based on the V̇O_2_max obtained in the three maximal incremental tests (unfatigued and after the 90 and 120 min runs), and the V̇O_2_ measured at 15, 90, and 120 min during the prolonged runs, to account for dynamic changes of both variables. Both variables were expressed relative to BM, recorded before and after the prolonged runs (i.e., before the step test) for trial V̇O_2_, and after the step test for V̇O_2_max. FU_run_ was then calculated as trial V̇O_2_/V̇O_2_max.

### 2.4 Statistical Analysis

All data are presented as mean ± SD. The Greenhouse–Geisser correction was applied when the assumption of sphericity was violated. A preliminary analysis involved comparing the physiological response during the prolonged runs up to the 90 min time point to confirm if, as expected, there was no difference in the physiological responses between the two runs to that point. This involved a two‐way repeated measure analysis of variance (ANOVA; main effect of condition and condition × time effect) for RE (expressed as EC and OC), BLa, HR, RPE, ventilation (V̇E), and respiratory exchange ratio (RER). Subsequently, to assess physiological changes of these variables throughout 120 min of running, a one‐way repeated measure ANOVA was used. BM changes from pre to post each prolonged run were analyzed via a paired *t*‐test, and BM measured before each incremental step test was assessed as a one‐way ANOVA.

Differences in maximal physiological responses to the V̇O_2_max ramp tests in the three conditions (unfatigued and post 90/120 min) were assessed via a one‐way ANOVA (condition effect). The physiological variables analyzed were: V̇O_2_max (in L·min^−1^ and mL·kg^−1^·min^−1^), sV̇O_2_max, BLa, RER, V̇E, and FU_run_. Physiology‐related measures corresponding to LT and LT2, specifically V̇O_2_, fractional utilization of V̇O_2_max, HR, RER, V̇E, and RPE, were calculated based on a linear regression between speed and the given measure during the step tests in each condition (unfatigued and post 90/120 min). The measures were then analyzed via a one‐way repeated measure ANOVA to assess the effect of prolonged running (condition effect). Post hoc analysis with Bonferroni adjustment was performed to identify the origin of any significant difference. To calculate differences in the rate of change between performance determinants, the percentage change from unfatigued at 90 and 120 min was analyzed with a two‐way repeated‐measure ANOVA. The variables considered were RE (expressed as OC), V̇O_2_max (mL·kg^−1^·min^−1^), and the FU_LT_, and their change was compared based on their effect on marathon performance speed (i.e., impairing or enhancing). Effect sizes were calculated as partial‐eta squared (η_p_^2^) and quantified as small (0.01–0.06), medium (0.06–0.14), and large (> 0.14). The threshold for significance was fixed at *p < 0.05*. All statistical analyses were performed using SPSS version 28 (SPSS Inc., Chicago, IL, USA).

## 3 Results

### 3.1 Participants and Trial Characteristics

Of the 17 participants who began at least one prolonged run, 14 completed the study. One participant could not complete the runs due to premature exhaustion, and 2 runners completed only one run but could not start the fatigued incremental test due to calf muscle discomfort. In addition, 1 participant completed both runs but was unable to start the step test following the 2 h trial; therefore, their data related to that were also excluded from the analysis. A summary of participant characteristics at baseline is shown in Table 1. Participants completed the prolonged runs at 14.1 ± 0.9 km·h^−1^, at an initial intensity corresponding to 79.3% ± 4.5% V̇O_2_max, covering 21.2 ± 1.4 km (range 19.5–24.5 km) and 28.2 ± 1.8 km (range 26.0–33.0 km) over the 90 min and 2 h trials, respectively. The physiological responses to the two prolonged runs were not different up to the 90 min time point, with no main effect of condition or condition × time effect found for RE, Bla, HR, V̇E, and RER (*p* > 0.19), confirming the consistency of the two trials up to this (90 min) point. Therefore, only data for the 120 min run are presented for these physiological responses to prolonged running. A time effect was found for BM (*F* = 35.45, *p* < 0.001, η_p_^2^ = 0.75), which from pretrial (68.7 ± 7.8 kg) decreased post 90 and 120 min (67.1 ± 7.6 and 66.8 ± 7.4 kg, respectively; *p* < 0.001 between every condition).

**TABLE 1:** Participant characteristics (*n* = 14).

Age (year)	Height (m)	Body mass (kg)	Mar. time (hh:mm:ss)	Mar. speed (km·h^−1^)	T. volume (km·w^−1^)	V̇O_2_max (mL·kg^−1^·min^−1^)	Trial speed (km·h^−1^)
35 ± 8	1.76 ± 0.08	68.7 ± 7.8	2:46:58 ± 0:10:37	15.2 ± 0.9	79 ± 23	63.1 ± 5.8	14.1 ± 0.9

*Note:* Data are expressed as mean ± SD.

Abbreviations: Mar. time/speed, best marathon time/speed in the 6 months before Visit 1; T. volume, training volume; V̇O_2_max: maximal oxygen uptake.

### 3.2 Effect of Run Duration on V̇O_2_peak

**FIGURE 2:**
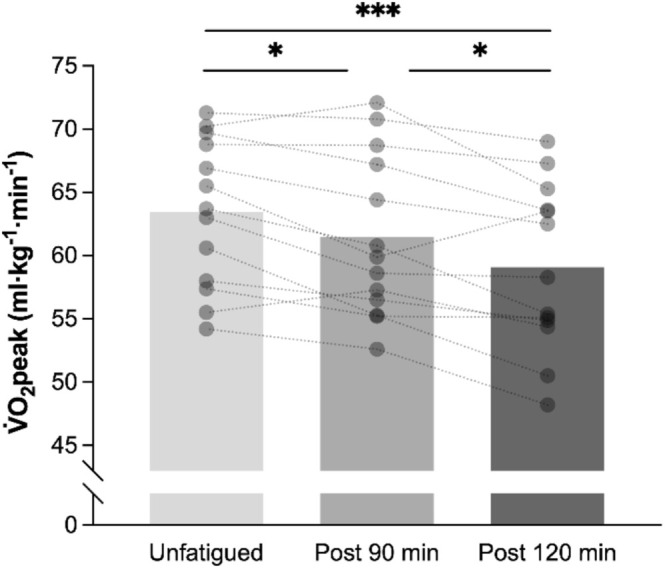
Changes in V̇O_2_peak (relative to body mass) reached at the end of the maximal ramp test in an unfatigued state and after 90 and 120 min of running in the heavy intensity domain. V̇O_2_peak calculations were adjusted for body mass changes following the 90/120 min run and step test. Columns indicate the mean for each condition (*n* = 13), and open circles the individual responses. *p < 0.05, ***p < 0.001.

In the maximal ramp tests to exhaustion, a V̇O_2_ plateau was found in 13, 7, and 2 participants (out of 13) in the unfatigued, post‐90, and post‐120 min trials, respectively. V̇O_2_peak was therefore used to describe maximal aerobic rate. When expressed relative to BM, V̇O_2_peak was −3.1% ± 3.7% lower post 90 min (*p* = 0.04) and −7.1% ± 4.5% lower post 120 min (*p* < 0.001; Figure 2 and Table 2) relative to unfatigued. Absolute V̇O_2_peak was −5.4% ± 3.2% lower after 90 min (*p* < 0.001) and −9.6% ± 4.7% lower after 120 min (*p* < 0.001; Table 2 and Figure S1 in online resources) compared to unfatigued. Results from the maximal ramp test indicated that estimated sV̇O_2_peak was reduced after 90 (−5.8%, *p* < 0.001) and 120 min (−10.7%, *p* < 0.001; Table 2), with HR max also lower compared to unfatigued (90 min: *p* = 0.008; 120 min: *p* < 0.001; Table 2). Similarly, BLa, RER, and V̇E at the end of the maximal ramp test decreased after 90 and 120 min (*p* < 0.001; Table 2 and Figure S1 in online resources).

**TABLE 2:** Physiological changes at V̇O_2_peak and lactate threshold (LT) before and after the 90 and 120 min runs (*n* = 13).

		Unfatigued	Post 90 min	Post 120 min	η_p_^2^
V̇O_2_peak	V̇O_2_ (mL·kg^−1^·min^−1^)	63.4 ± 5.9	61.5 ± 6.5*	59.0 ± 6.7**^,#^	0.60
	V̇O_2_ (L·min^−1^)	4.34 ± 0.57	4.12 ± 0.61**	3.93 ± 0.53**^,#^	0.73
	Speed (km·h^−1^)	18.1 ± 1.1	17.1 ± 1.2**	16.2 ± 1.0**^,#^	0.76
	BLa (mMol·L^−1^)	7.7 ± 1.3	3.7 ± 1.1**	2.9 ± 0.9**^,#^	0.90
	HR max (beats·min^−1^)	182 ± 5	178 ± 6*	175 ± 5**^,#^	0.81
	RER	1.06 ± 0.03	1.01 ± 0.06**	0.94 ± 0.05**^,#^	0.79
	V̇E (L·min^−1^)	126 ± 20	117 ± 17*	106 ± 16**^,#^	0.66
LT	Speed (km·h^−1^)	14.0 ± 0.9	13.5 ± 1.0*	13.0 ± 1.1**^,##^	0.80
	V̇O_2_ (mL·kg^−1^·min^−1^)	49.8 ± 4.6	49.3 ± 5.0	48.8 ± 4.7	0.21
	HR (bpm·min^−1^)	154 ± 6	157 ± 5*	157 ± 5	0.38
	RER	0.95 ± 0.04	0.91 ± 0.03	0.89 ± 0.03**^,#^	0.61
	V̇E (L·min^−1^)	80 ± 15	79 ± 14	77 ± 14	0.25
	RPE (6–20)	13.1 ± 0.9	14.0 ± 0.9	14.7 ± 1.1*	0.48

*Note:* Physiological parameters in column 2 refer to the performance determinant in column 1. *p < 0.05 **p < 0.001 vs. unfatigued; # *p* < 0.05 ## *p* < 0.001 vs. post 90 min. Effect size (η_p_^2^) values > 0.14 were considered “large”.

Abbreviations: BLa, blood lactate; HR, heart rate; LT, lactate threshold; RER, respiratory exchange ratio; RPE, rate of perceived exhaustion; V̇E, ventilation; V̇O_2_peak, peak oxygen uptake; η_p_^2^, effect size as partial eta squared.

### 3.3 Changes in RE, FU_run_ , and Physiology During the 120 Min Prolonged Run

**FIGURE 3:**
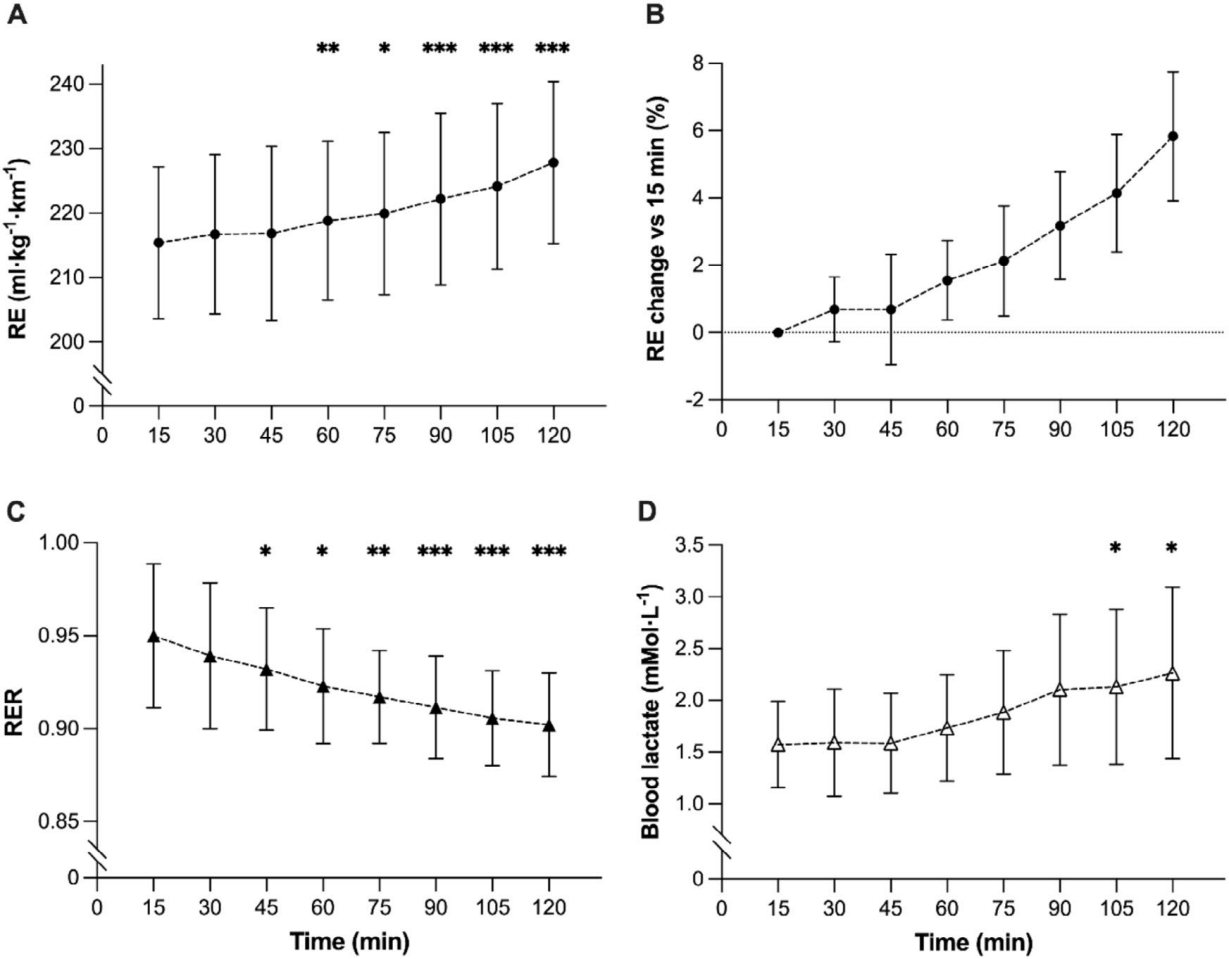
Changes in physiology during the 120 min running trial. Running economy (solid circles, A); the percentage change of RE relative to the initial 15 min time point (B); respiratory exchange ratio (solid triangles, C); Blood lactate concentration (open triangles, D). Different from 15 min: *p < 0.05, **p < 0.01, ***p < 0.001. Data are mean ± SD (*n* = 14).

**FIGURE 4:**
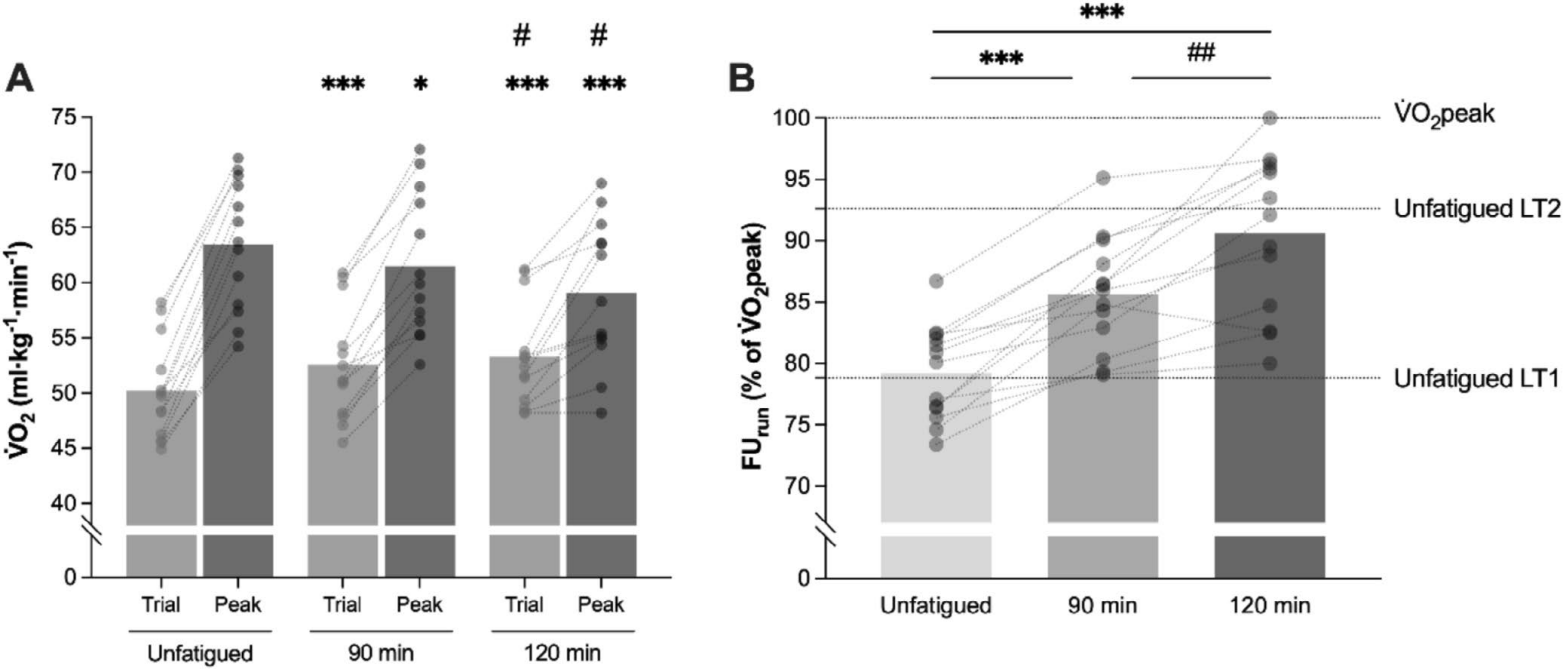
Panel A shows V̇O_2_ (light gray column) at 15 (unfatigued), 90, and 120 min during the prolonged run, and V̇O_2_peak (obtained during maximal ramp tests to exhaustion; dark gray column) in an unfatigued state, and post 90 and 120 min of prolonged running. Panel B shows the changes in fractional utilization during the prolonged run, expressed as % of V̇O_2_peak, in the three conditions. The dotted horizontal lines indicate the mean % V̇O_2_peak corresponding to LT and LT2 in an unfatigued state. Columns indicate the mean for each condition (*n* = 13), and circles indicate individual responses. Difference from unfatigued: *p < 0.05, **p < 0.01, ***p < 0.001; Difference between 90 and 120 min: # *p* < 0.05, ## *p* < 0.01.

During the 120 min run, a main effect of time was present for RE (*F* = 65.21, *p* < 0.001, η_p_^2^ = 0.83), with significant differences between 15 min (215 ± 12 mL·kg^−1^·km^−1^) and 60 and 75 min (*p* = 0.01). RE continued to deteriorate at 90, 105, and 120 min, reaching +5.8% ± 1.9% (228 ± 13 mL·kg^−1^·km^−1^, *p* < 0.001; Figure 3) compared to 15 min. FU_run_ increased with run duration from 79.3% ± 4.5% (unfatigued) to 85.6% ± 4.7% (post 90) and 90.6 ± 6.5*%* of V̇O_2_peak (post 120 min; all *p* < 0.001), due to the increase in submaximal V̇O_2_ and decrease in V̇O_2_peak (Figure 4).

A large time effect was found for RER (*F* = 29.3, *p* < 0.001, η_p_^2^ = 0.69), with changes from 15 min significant after 45 min (*p* = 0.02) and a reduction of 5.0% ± 2.9% by the end of the 120 min trial (*p* < 0.001; Figure 3). Similarly, a significant time effect was found for BLa (*F* = 26.92, *p* < 0.001, η_p_^2^ = 0.67), with an increase from 15 min at 105 and 120 min (*p* = 0.02; Figure 3). There was also a large time effect for HR (*F* = 36.34, *p* < 0.001, η_p_^2^ = 0.74), RPE (*F* = 49.74, *p* < 0.001, η_p_^2^ = 0.88), and V̇E (*F* = 13.88, *p* < 0.001, η_p_^2^ = 0.52). Data and post hoc analysis are reported in Figure 3 and online resources (Table S1).

### 3.4 Effect of Run Duration on Lactate Threshold

**FIGURE 5:**
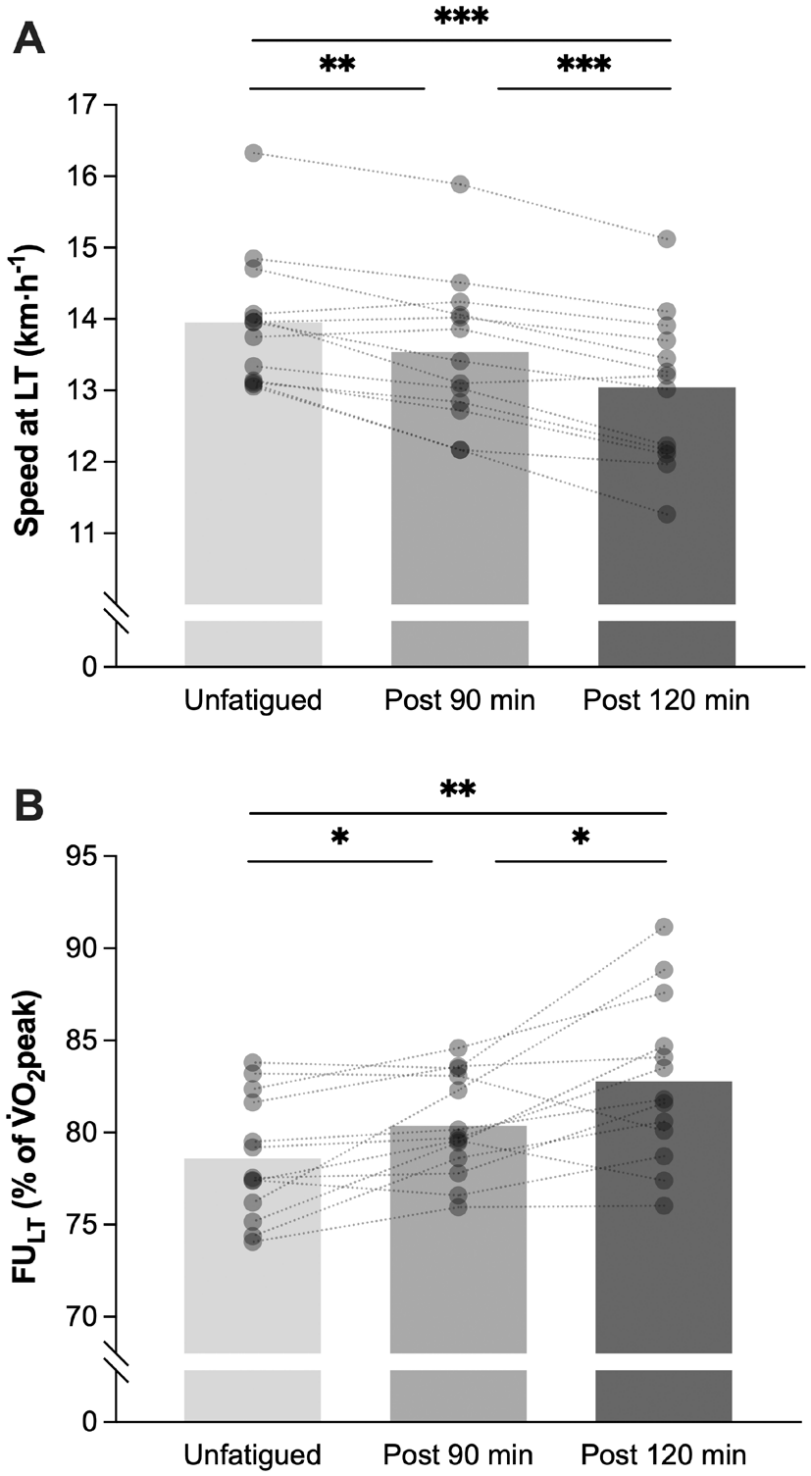
Changes in speed at LT (A) and fractional utilization of V̇O_2_peak at LT (FU_LT_; B), in an unfatigued state, and after 90 and 120 min of running. Columns indicate the mean for each condition (*n* = 13), and open circles the individual responses. *p < 0.05, **p < 0.01, ***p < 0.001.

sLT showed a decrease from 14.0 ± 0.9 in an unfatigued state to 13.5 ± 1.0 km·h^−1^ after 90 min (−3.0%, *p* = 0.005), and to 13.0 ± 1.1 km·h^−1^ after 120 min (−6.6%, *p* < 0.001; Figure 5). FU_LT_ increased with accumulated running time from 78.6% ± 3.3% (unfatigued) to 80.4% ± 2.8% of V̇O_2_peak after 90 min (+2.2%; *p* = 0.03), and further to 82.8% ± 4.5% after 120 min (+4.9%; *p* = 0.01; Figure 5). A condition effect was also present for other physiological markers corresponding to sLT as follows: increased HR (*p* = 0.01) and RPE (*p* < 0.001); decreased RER (*p* < 0.001) and V̇E (*p* = 0.03), while V̇O_2_ relative to BM remained unchanged (*p* = 0.06). Post hoc analyses are displayed in Table 2.

Due to the low concentration of BLa in the step test following the prolonged runs, it was not possible to determine LT2. An example of a participant's lactate curve in the three conditions is provided in Figure S2 in online resources. In an unfatigued state, the speed and V̇O_2_ at LT2 were 16.7 ± 1.1 km·h^−1^ and 4.03 ± 0.55 L·min^−1^, corresponding to 92.6% ± 3.3% of V̇O_2_max.

### 3.5 Comparison of Changes in Physiological Determinants

**FIGURE 6:**
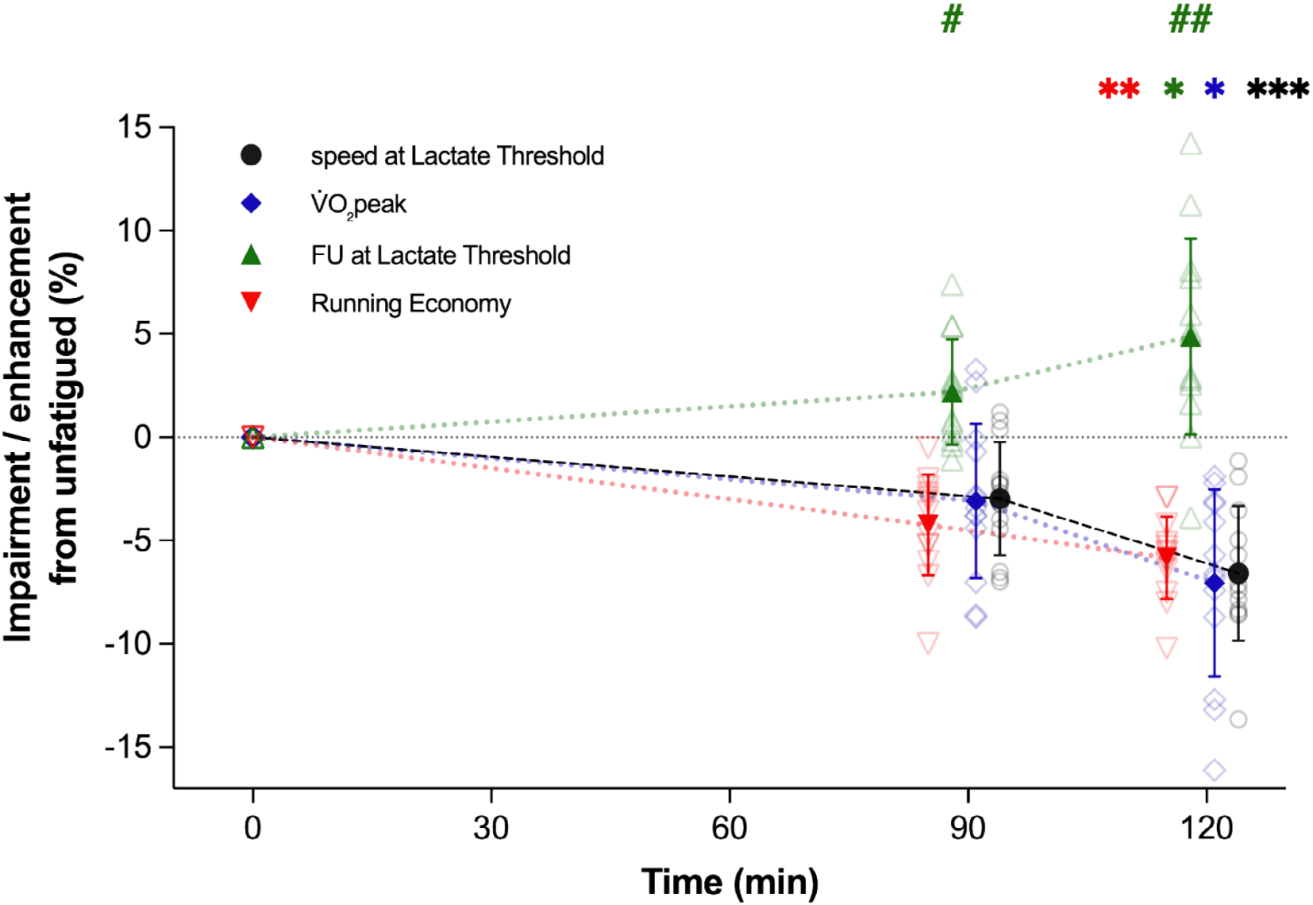
Changes in speed at lactate threshold (black circles) and endurance performance determinants: V̇O_2_peak (blue diamonds), fractional utilization of V̇O_2_peak at lactate threshold (green triangles), and running economy (red triangles); after 90 and 120 min of running in the heavy intensity domain. Open symbols indicate individual responses. Asterisk colors denote the significance of the corresponding variable. Different from 90 min: *p < 0.05, **p < 0.01, ***p < 0.001. Change from unfatigued different than other performance determinants: # *p* < 0.05, ## *p* < 0.01. Data are mean ± SD (*n* = 13).

Following the prolonged runs, FU_LT_ was found to increase by 2.8% (90 min) and 4.9% (120 min), compared to unfatigued. RE deteriorated by 4.2% (90 min) and 5.8% (120 min), and V̇O_2_peak decreased by 3.1% (90 min) and 7.1% (120 min). When the magnitude of these changes from unfatigued to post‐90 and post‐120 min was compared between physiological determinants, to assess their impact on marathon performance speed, a determinant × condition interaction effect was found (*F* = 17.45, *p* < 0.001, η_p_^2^ = 0.59). Post hoc analysis revealed that FU_LT_ changed differently than V̇O_2_max and RE after 90 (*p* = 0.02) and 120 min (*p* = 0.001 vs. V̇O_2_max; *p* < 0.001 vs. RE). Figure 6 displays the changes in physiological determinants and their potential effect (impairing or enhancing) on marathon performance. sLT is included in Figure 6 as a proxy of marathon speed, given the close association between the two.

## 4 Discussion

This study aimed to assess the fatigue‐induced changes to the determinants of endurance running performance and sLT after running for 90 and 120 min in the heavy‐intensity domain in well‐trained male marathon runners. The results demonstrate for the first time that all three performance determinants change after 90 min of running and are further altered between 90 and 120 min. Specifically, RE showed a relatively linear deterioration (by −4.2% and −5.8% at 90 and 120 min), V̇O_2_peak decreased in an increasingly nonlinear manner, subtly at 90 min, but more markedly after 120 min (by −3.1% and −7.1%), whereas FU_LT_ increased by +2.8 and +4.9%. sLT, which is closely associated with marathon speed, was found to decrease by 3.0% and 6.6% after 90 and 120 min. Moreover, the simultaneous decrease in V̇O_2_peak and deterioration in RE led to a large increase in trial intensity (i.e., FU_run_), from 79 to 85 and 91% V̇O_2_peak between 15, 90, and 120 min, making the prolonged run increasingly unsustainable and explaining the development of fatigue.

### 4.1 V̇O_2_Peak

This is the first study to report duration‐dependent changes in V̇O_2_peak following prolonged running, with an accelerating decline (~4‐fold greater rate) between 90 and 120 min (+4%) compared to 0–90 min of running (+3%). These results are somewhat similar to a reported decrease (6%–7%) in V̇O_2_max following a half‐marathon race [13]. Given the nonlinear deterioration of V̇O_2_peak and the relatively small change at 90 min, the results may also align with a recent report of no changes after a 60 min run in trained runners [12]. During cycling, V̇O_2_peak has been found to be unchanged after 2 h of heavy intensity exercise [6, 18] but decreased by 6% after 90 km of cycling [16]. Collectively, these data suggest an accelerating duration‐dependent decrease in V̇O_2_peak after prolonged exercise. The decrease in sV̇O_2_peak (−10.7% post 120 min) was more pronounced than V̇O_2_peak due to a deterioration in RE, which is similar to the power output reduction at V̇O_2_peak reported after 3 h of cycling (−9%) [16]. Several mechanisms may explain the decreased V̇O_2_peak after 120 min of running. Firstly, the reduction in HRmax during the fatigued V̇O_2_peak tests suggests cardiac output was reduced at maximal intensity [32]. Cardiac output could have also been reduced due to a decline in stroke volume, evidenced by the upward drift in HR during the prolonged run, likely caused by an increase in core temperature and dehydration [33]. These changes may have impaired O_2_ delivery to the working muscles and therefore reduced V̇O_2_peak. In the current study, we also found large decreases in BLa and RER during the fatigued ramp tests, greater than previous research of similar exercise duration (running [13] cycling [16] cross‐country skiing [34]). This may have been due to the higher intensity of our trial than previous investigations (~80% vs. 50%–70% V̇O_2_peak) leading to greater glycogen depletion and impaired anaerobic glycolysis [35], which indirectly appeared low considering the limited increase in BLa concentrations (2.9 mMol·L^−1^) after the ramp test at 120 min. As muscle glycogen is the primary substrate for oxidative metabolism during intense exercise, glycogen depletion could also partially explain the reduction in V̇O_2_peak, due to limited glucose available for energy production. Finally, the failure to achieve a plateau in V̇O_2_ at the end of the maximal ramp test in 6 and 11 participants (out of 13) after 90 and 120 min, respectively, may imply that several athletes did not terminate the test due to a limitation in V̇O_2_, but because of other factors.

### 4.2 Running Economy

The deterioration of RE (expressed as OC) during this experiment (+4.2% and +5.8% at 90 and 120 min) is consistent with previous studies of similar duration and distance [8, 36], and in line with results from a recent study from our group at a similar intensity [11]. Compared to OC, EC deteriorated in a similar manner and slightly lower rate (+4.0% and +5.3% at 90 and 120 min) due to the decrease in RER during the runs (i.e., shift to fat oxidation tending to elevate OC more than EC). This small difference between OC and EC seems unlikely to significantly impact the durability of RE, as previously demonstrated in a large cohort of runners of similar characteristics [11]. This deterioration is likely due to the activation of less efficient type II fibers, as type I muscle fibers become glycogen depleted during prolonged exercise [37]. Biomechanical alterations have also been observed as fatigue increases [38], with a shift in the work performed by the ankle plantar flexors towards musculature around the knee and hip, which may contribute to changes in RE after exhaustive prolonged runs [39]. Electromyographic (EMG) measurements during marathon running have also revealed heightened neural input to the muscles in a fatigued state, which has been linked to a deterioration of RE [10]. Altered activation patterns likely suggest a larger number of motor units are recruited, leading to a deterioration in RE [40]. A reduction in leg stiffness has also been reported following high‐intensity running [41] which could further contribute to a deteriorated RE, due to lesser elastic energy storage and release during the stretch‐shortening cycle. Future studies could therefore investigate the mechanisms underlying RE changes during prolonged running. Moreover, the RER decrease from the start to the end of the run in this study indicates a higher reliance on fats as a source of energy as the run progresses, which are less metabolically efficient than CHO and require greater V̇O_2_ to generate a given amount of energy [31].

### 4.3 Fractional Utilization During the Run and at the Lactate Threshold

An increased fractional utilization was found during the prolonged run (FU_run_), increasing from 79% (15 min) to 86% (90 min) and 91% of V̇O_2_peak (120 min). The marked increase in FU_run_ equated to an increase in exercise intensity from the bottom to the top of the heavy domain over the 2 h run (Figure 4). Remarkably, four individuals were likely exercising in the severe intensity domain at the end of the 2 h run, reaching a FU_run_ > 95% of V̇O_2_peak after 120 min, well above their % of V̇O_2_peak at LT2 in the unfatigued state. In contrast to these results, after 2 h of cycling fractional utilization was found to increase more modestly 65% vs. 68% V̇O_2_peak [6, 17], due in part to the unchanged V̇O_2_peak. To the author's knowledge, this study is the first to report changes in FU_LT_ after different durations of prolonged running. The results revealed a nonlinear pattern, with a modest increase from the unfatigued state to 90 min (+2.2%), and a more rapid change from 90 to 120 min (+4.9% vs. unfatigu0ed). This increase appears to contrast with a recent study reporting a 5% decrease in FU_LT_ following prolonged cycling [7]; however, in that study V̇O_2_max was assumed to remain constant throughout the exercise, and thus the findings are not comparable to the current investigation.

Both measures of fractional utilization exhibited an accelerating curvilinear increase, with a greater increase in FU_run_ (+12% V̇O_2_peak after 120 min) due to being measured at a fixed speed, compared to a modest increase in FU_LT_ (+5% V̇O_2_peak), due to a decline in sLT (−1.0 km·h^−1^). V̇O_2_ at LT remained unchanged, likely due to the deterioration of RE (+5.8% after 120 min) counteracting the reduced sLT. Consequently, the observed increase in FU_LT_ likely primarily reflect the decline in V̇O_2_peak, representing a diminished physiological reserve at heavy exercise intensity, rather than an improvement in the fraction of aerobic capacity that is accessible for the exercise duration. The concurrent changes in V̇O_2_peak and RE also explain the rise in FU_run_ from 15 to 120 min, both contributing to the increase. The current study is the first to show the dynamic nature and interplay of the decrease in V̇O_2_peak and rise in fractional utilization following prolonged running at marathon‐like intensity.

### 4.4 Lactate Threshold

The effect of prolonged running on sLT led to a progressive nonlinear decrease from 14.0 to 13.5 (−3.0%) and 13.0 km·h^−1^ (−6.6%) after 90 and 120 min. Similar to this study, alterations of the moderate‐heavy domain boundary have recently been found in cycling, with an 8%–10% reduction after 150–180 min [7, 16]. A nonlinear change in power output at VT1 was also reported during moderate‐intensity exercise in cycling, with no decrease at 60 min and an estimated 5% decrease after ~140 min [21]. The consistent V̇O_2_ at sLT across the conditions (unfatigued, post‐90, and post‐120 min) of the current study is in contrast to some recent reports showing a decline in V̇O_2_ at the moderate‐heavy threshold following prolonged cycling (−3% to 7%) [7, 16], possibly due to a different unit used to express V̇O_2_, per BM here and absolute in previous studies. HR at LT marginally increased (+2 beats·min^−1^) after 120 min in the present study, similar to Bitel et al. [16], whereas a higher increase has been found by Stevenson et al. [7], possibly due to differences in the postexercise timing of the step test. The post‐120 min changes in V̇E and RPE at sLT were similar or larger than in longer cycling studies [7, 16], which may be due to differences in the intensity domain of the exercise—heavy in this study and (predominantly) moderate in other investigations [7, 16].

### 4.5 Potential Effects on Marathon Performance

The effect of physiological determinants changes following prolonged running on marathon performance differed, with the deterioration of V̇O_2_peak and RE tending to impair performance, whereas the increase in FU_LT_ after both 90 and 120 min would, based upon Joyner's model, limit the decline in performance. Interestingly, alterations in V̇O_2_peak and FU_LT_ were particularly marked between 90 and 120 min of running, while RE changes were more linear, indicating that performance determinants may change at different rates during prolonged running. Similarly, sLT was reduced in a nonlinear manner, which may also be the case for sustainable marathon speed, given the close association between sLT and marathon performance speed [2, 3]. Due to the small sample size of this study, an analysis to identify the potential impact of each physiological determinant on marathon performance was not possible, and this could be an interesting avenue for future research. From unfatigued to 120 min, large between‐participants differences were found for changes in each performance determinant (0%–16%) and sLT (1%–14%). The individual differences found despite the similar training and racing background of the participants highlight the importance of measuring fatigued physiology to inform marathon performance. Given this variability in individual responses, it would be of interest to investigate associations between the durability of physiological determinants (and sLT) and marathon performance, which may confirm the importance of assessing durability for race predictions. It seems intuitive that sLT (and speed at LTP) durability may best reflect marathon performance, considering the close association between the two parameters in highly trained and elite athletes [1, 2]. The individual variations found are in line with previous studies, reporting a 2%–14% decrease in time trial performance [42] and a 1%–32% reduction in critical power [17] after prolonged cycling. It is also worth noting that the fixed speed of the prolonged run and the limited CHO intake provided may have influenced the physiological changes observed, and in a real‐world scenario (i.e., during a marathon) a smaller decay may occur.

### 4.6 Implications for Training and Performance

The changes in V̇O_2_peak, RE, and sLT may have important implications for our understanding of performance and training prescription, typically based on measurements in an unfatigued state. Despite including well‐trained marathon runners in this study, the trial intensity (FU_run_) drifted upwards from the bottom to the top of the heavy intensity domain during the 2 h run. A further drift in intensity may lead to exercise in the severe domain, which would rapidly cause exhaustion and may promote different physiological adaptations to those produced when exercising in the heavy domain [15, 43]. This is important to consider for long‐duration training sessions starting close to the heavy–severe domain boundary, such as “tempo runs”. A potential strategy to ensure exercise intensity remains constant during prolonged exercise may be the use of fixed HR, given the very limited drift found in HR corresponding to the moderate–heavy domain boundary. However, in this study we were unable to assess changes in LT2; therefore, further investigation is warranted to measure fatigue‐dependent changes in physiological parameters in correspondence to LT2. The similar decline in sLT from unfatigued to 90 vs. from 90 to 120 min (+3% vs. +3.6%) indicates that for races within the heavy intensity domain, longer events such as the marathon may be particularly affected by durability. This is supported by the nonlinear change in V̇O_2_peak and FU_LT_, implying that several physiological variables may remain relatively unchanged for a certain running duration but then increasingly deteriorate as fatigue accumulates. It is therefore of interest for future research to (a) explore possible strategies to enhance durability—e.g., strength training, already proven effective in several endurance sports [44, 45, 46]—and (b) investigate a larger cohort of athletes to model marathon performance based on the integration of unfatigued physiology and durability. These findings could subsequently improve marathon performance.

### 4.7 Limitations

This study is not without limitations. First, although the conditions closely reproduced the intensity of a marathon race, the duration was limited to 2 h (33 km for the fastest runner); thus, it is difficult to predict how physiological parameters may have behaved beyond this time point. Exercising beyond 2 h would, however, have been very challenging for participants, given the high level of RPE (16.4 ± 1.9) and fractional utilization (90.6% ± 6.5%) reached at the end of the 120 min trial. Moreover, this study involved constant paced running during both trials without any speed fluctuation, contrary to marathon race conditions where athletes pacing shows subtle variation even on relatively flat courses. A design with higher ecological validity may have allowed for a longer trial and allowed some subtle variation in pacing. Secondly, the assessment timing of the performance determinants differed, with RE measured during the prolonged run and V̇O_2_peak and FU_LT_ measured at one time point in each session. This was done to not confound the response to the prolonged run. Although RE is best measured during the trial due to a possible speed‐dependent change in RE durability [9, 22], this is not possible for LT and V̇O_2_max, as their evaluation at the beginning of the run would induce fatigue early on during the run. A third limitation relates to the amount of CHO ingested during exercise (30 g·h^−1^). Well‐trained marathon runners are typically recommended to ingest CHO at a higher rate in competition than was used in the current study [47], which may better preserve muscle glycogen and offset the fatigue‐induced changes we have observed. However, this approach may have led to gastrointestinal discomfort in runners not accustomed to high CHO intake; hence why a lower CHO rate was preferred. Finally, this research only included male participants, and consequently, the results cannot be extrapolated to female runners. Only males were included due to the currently unknown effect of menstrual cycle phase on the durability of endurance performance determinants. Although menstrual cycle phase could have been standardized, this would have required the prolonged runs to be spaced by approximately 1 month to account for phase‐specific variations. Such a design could have introduced training‐induced adaptations, confounding the interpretation of the results. Additionally, if female participants had been included and matched to males based on performance level (e.g., World Athletics points), they would have covered a substantially shorter distance over the prolonged runs, which has an impact on durability as recently demonstrated [11].

## 5 Perspective

These findings represent the first evidence that all three physiological determinants of endurance running performance, and sLT, change in well‐trained endurance runners following 90 min of running in the heavy intensity domain, and are particularly altered after 120 min. Changes differed between determinants, with V̇O_2_peak and RE deteriorating by −7.1 and −5.8%, concomitant to an increase in FU_LT_ (+4.9%) primarily due to the reduction in V̇O_2_peak. Alterations in V̇O_2_peak and FU_LT_ were particularly marked between 90 and 120 min of running, while RE changed more linearly. sLT, which is closely associated with marathon speed, also decreased at an accelerating rate from 14.0 to 13.5 (post 90 min) to 13.0 km·h^−1^ (post 120 min). Furthermore, the decrease in V̇O_2_peak and the increase in RE during the prolonged run contributed to a substantial and nonlinear increase in FU_run_, from 79% to 91% of V̇O_2_peak between the run's start and end. These dynamic changes have strong implications for running performance, its modeling, and training, and are likely to particularly affect longer duration running events such as the marathon. Future investigations should focus on strategies to improve durability, which may subsequently enhance marathon performance, and test durability under conditions akin to a marathon race (e.g., advanced footwear technology, higher CHO intake, tapered training) to enhance ecological validity. Furthermore, research should investigate the durability of physiological determinants in female athletes and the effect of menstrual cycle phase on these parameters.

## Author Contributions

M.Z., R.C.B., and J.P.F. conceived the study; M.Z. collected the data; M.Z. analyzed the data and wrote the initial manuscript draft; M.Z., R.C.B., and J.P.F. contributed to the draft updates. All authors approved the final version.

## Ethics Statement

The study was approved by the Loughborough University Ethics Sub‐Committee and conducted in accordance with the Declaration of Helsinki. All participants provided written informed consent prior to participation.

## Conflicts of Interest

The authors declare no conflicts of interest.

## Supporting information


Data S1.

## Data Availability

Data supporting the results presented in the manuscript are included in the figures and online resources whenever possible, and are available upon request to the corresponding author.
