# Supplementary material for: The Effect of 90 and 120 Min of Running on the Determinants of Endurance Performance in Well‐Trained Male Marathon Runners
Source: Scand J Med Sci Sports. 2025 May 15;35(5):e70076. doi: 10.1111/sms.70076 (PMC12082016; doi:10.1111/sms.70076)
Supplement: Supplementary file 1 — Data S1. [file SMS-35-e70076-s001.docx]

**Supplementary Materials**

**The effect of 90 and 120 min of running on the determinants of endurance running performance in well-trained male athletes**

**Journal:** Scandinavian Journal of Medicine and Science in Sports

**Authors:**

Michele Zanini^1^, Jonathan P. Folland^1,2^, Richard C. Blagrove^1^

1. School of Sport, Exercise, and Health Sciences; Loughborough University (UK)

2. National Institute for Health and Care Research (NIHR); Leicester Biomedical Research Centre, Leicester (UK)

**Corresponding author:**

Michele Zanini (ORCID: 0009-0007-8148-8843)

School of Sport, Exercise & Health Sciences

Loughborough University, LE11 3TU, United Kingdom

E-mail: [m.zanini@lboro.ac.uk](mailto:m.zanini@lboro.ac.uk)

**Table S1.** Physiological changes during the 120 min prolonged run (n=14).

|  | **Prolonged run time (min)** | | | | | | | |
| --- | --- | --- | --- | --- | --- | --- | --- | --- |
|  | **15** | **30** | **45** | **60** | **75** | **90** | **105** | **120** |
| OC (ml·kg^-1^·km^-1^) | 215 ± 12 | 217 ± 12 | 217 ± 13 | 219 ± 12* | 220 ± 13* | 222 ± 14** | 224 ± 13** | 228 ± 12** |
| EC (kcal·kg^-1^·km^-1^) | 1.08 ± 0.06 | 1.08 ± 0.06 | 1.08 ± 0.07 | 1.09 ± 0.06* | 1.09 ± 0.06* | 1.1 ± 0.07** | 1.11 ± 0.06** | 1.13 ± 0.06** |
| HR  (bpm·min^-1^) | 155 ± 6 | 158 ± 6* | 160 ± 6* | 161 ± 7* | 162 ± 7* | 164 ± 7** | 165 ± 7** | 166 ± 7** |
| RER | 0.95 ± 0.04 | 0.94 ± 0.04 | 0.93 ± 0.03* | 0.92 ± 0.03* | 0.92 ± 0.02* | 0.91 ± 0.03** | 0.91 ± 0.03** | 0.90 ± 0.03** |
| V̇E  (L·min^-1^) | 82 ± 15 | 83 ± 15 | 84 ± 15 | 84 ± 15 | 85 ± 16 | 85 ± 16 | 87 ± 16 | 89 ± 17* |
| BLa  (mMol·L^-1^) | 1.57 ± 0.4 | 1.59 ± 0.5 | 1.59 ± 0.5 | 1.73 ± 0.5 | 1.89 ± 0.6 | 2.11 ± 0.7 | 2.13 ± 0.7* | 2.26 ± 0.8* |
| RPE | 11.2 ± 1.6 | 11.9 ± 1.4 | 12.9 ± 1.4** | 13.9 ± 1.5** | 14.8 ± 1.5** | 15.5 ± 1.9** | 15.9 ± 2.5** | 16.4 ± 1.9** |

OC: oxygen cost; EC: energy cost; HR: heart rate; RER: respiratory exchange ratio; V̇E: ventilation; BLa: blood lactate; RPE: rate of perceived exhaustion. * p<0.05 ** p<0.001 vs 15 min.

**
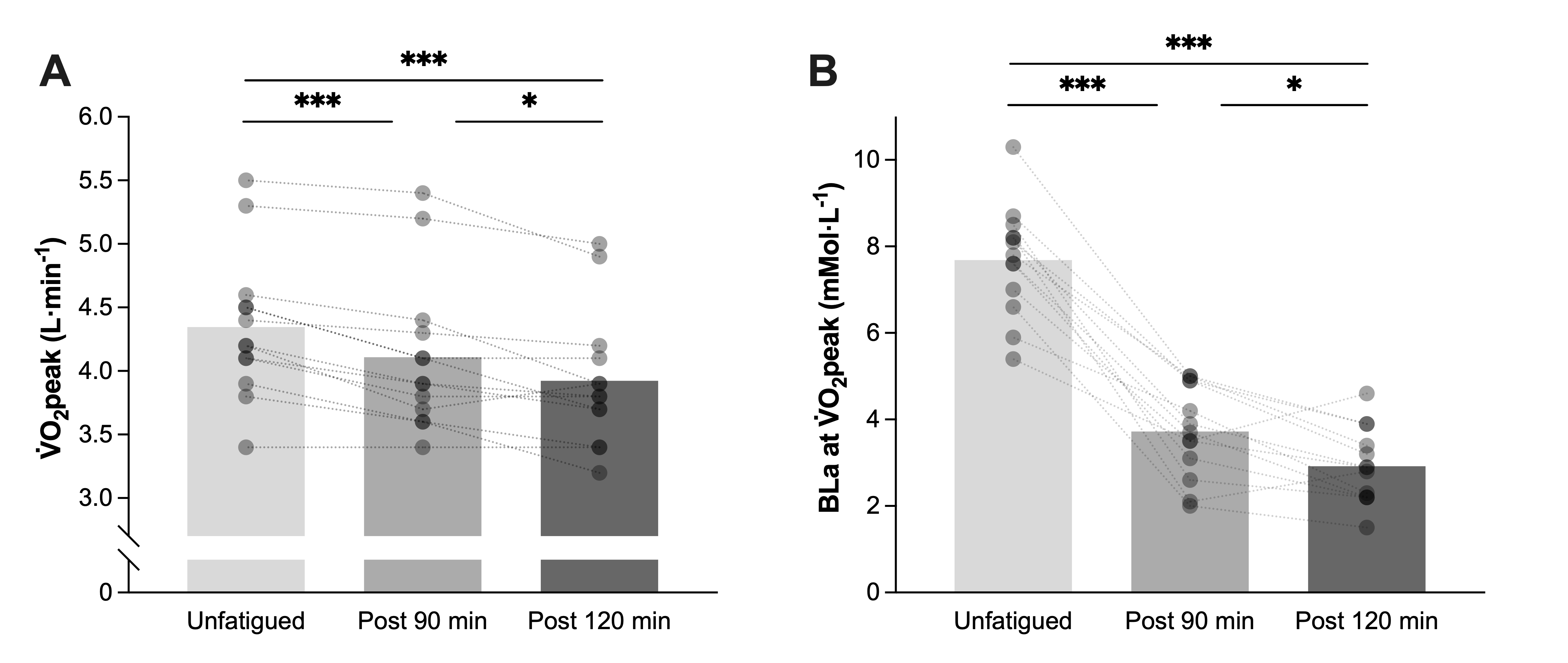
**

**Fig. S1.** Changes in absolute V̇O_2_peak (A) and peak blood lactate (B) reached at the end of the maximal ramp test, in an unfatigued state, and after 90 and 120 min of running in the heavy intensity domain. Columns indicate the mean for each condition (n=13), and open circles the individual responses. * p<0.05, *** p<0.001.

**
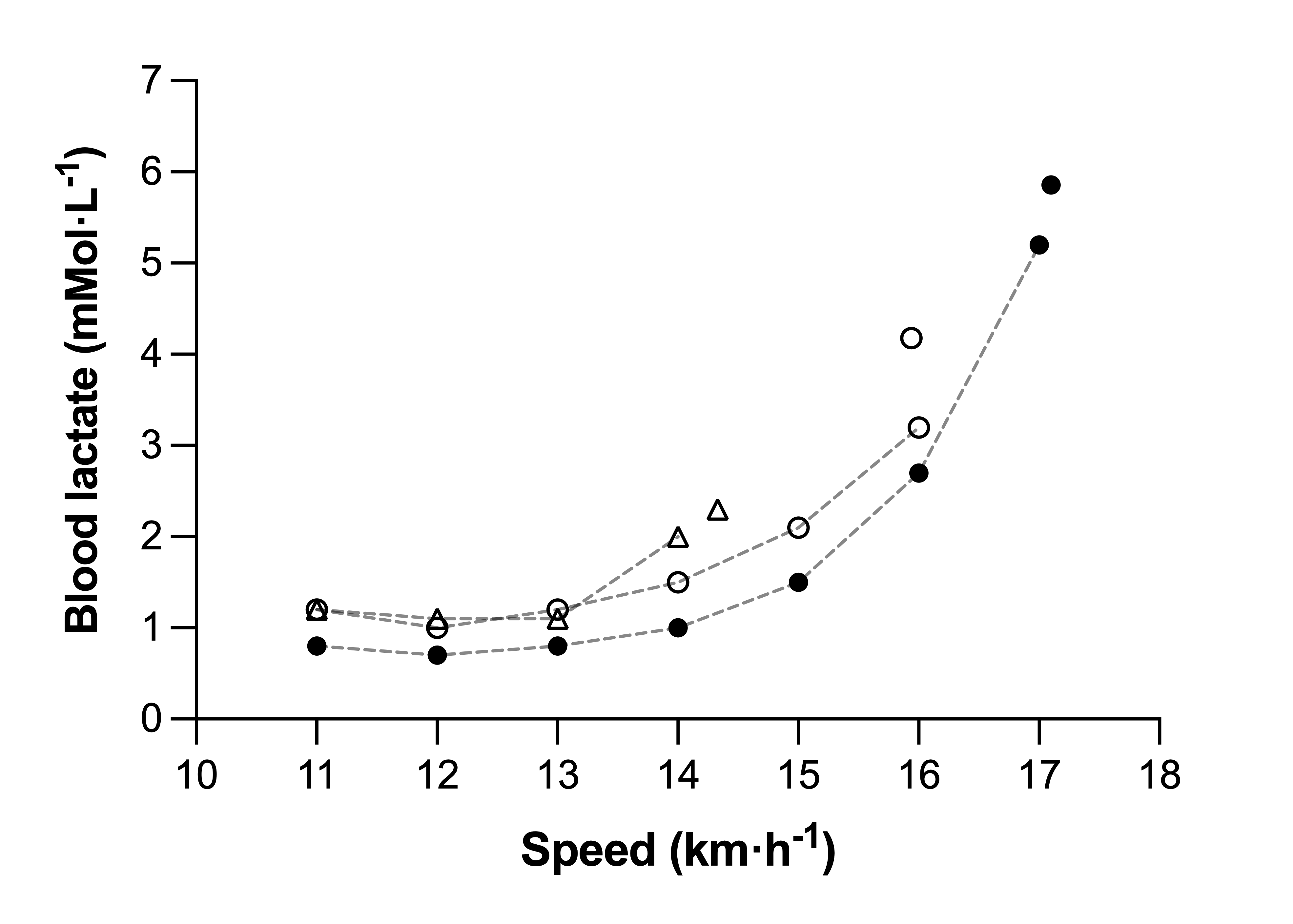
**

**Fig. S2.** Blood lactate response of a representative participant during the step test in the three conditions: unfatigued (solid circles), after 90 min (open circles), and after 120 min (open triangles) of running. Datapoints not connected by the dashed line indicate peak blood lactate during the maximal ramp test and estimated sV̇O_2_peak, for each condition.
